# Progression of Gene Expression Changes following a Mechanical Injury to Articular Cartilage as a Model of Early Stage Osteoarthritis

**DOI:** 10.1155/2014/371426

**Published:** 2014-11-16

**Authors:** R. S. McCulloch, M. S. Ashwell, C. Maltecca, A. T. O'Nan, P. L. Mente

**Affiliations:** ^1^Joint Department of Biomedical Engineering, North Carolina State University, Raleigh, NC 27695, USA; ^2^University of North Carolina, Chapel Hill, NC 27599, USA; ^3^Human Physiology Department, Gonzaga University, Spokane, WA 99258, USA; ^4^Department of Animal Science, North Carolina State University, Campus Box 7621, Raleigh, NC 27695, USA

## Abstract

An impact injury model of early stage osteoarthritis (OA) progression was developed using a mechanical insult to an articular cartilage surface to evaluate differential gene expression changes over time and treatment. Porcine patellae with intact cartilage surfaces were randomized to one of three treatments: nonimpacted control, axial impaction (2000 N), or a shear impaction (500 N axial, with tangential displacement to induce shear forces). After impact, the patellae were returned to culture for 0, 3, 7, or 14 days. At the appropriate time point, RNA was extracted from full-thickness cartilage slices at the impact site. Quantitative real-time PCR was used to evaluate differential gene expression for 18 OA related genes from four categories: cartilage matrix, degradative enzymes and inhibitors, inflammatory response and signaling, and cell apoptosis. The shear impacted specimens were compared to the axial impacted specimens and showed that shear specimens more highly expressed type I collagen (*Col1a1*) at the early time points. In addition, there was generally elevated expression of degradative enzymes, inflammatory response genes, and apoptosis markers at the early time points. These changes suggest that the more physiologically relevant shear loading may initially be more damaging to the cartilage and induces more repair efforts after loading.

## 1. Introduction

Osteoarthritis is estimated to affect 27 million Americans and this number is predicted to rise over the coming years [1]. While the causes and progression of OA are not completely understood, a prior joint injury is a known predisposing factor for the development of OA [2]. Therefore, in a laboratory setting, an injury model may be used to study the early stage progression of cartilage degeneration.

One common method of modeling OA in a laboratory setting is that of an impact injury. In this scenario, a controlled impact is delivered to the joint surface and induced changes are evaluated. The impacts can be done* in vivo* [3–5] or* in vitro *[6–9]. However, an* in vivo* impact injury may prove difficult to evaluate in terms of ongoing loading following the discrete loading event. Thus, an* in vitro* model allows for much more accurate quantification of the mechanical forces delivered to the articular surface. Most impact studies have utilized loading normal to the cartilage surface [3, 5–11]; however a real physiologic loading event likely has loading along multiple axes. Therefore one of our aims was to employ a more complex impact model with elevated shear loading.

Identifying differences in gene expression related to OA progression may aid in the identification of pathways of early stage disease development. Combining an impact injury model with an evaluation of gene expression changes may help to identify future targets for intervention during OA progression. Previous studies have utilized cyclical loading [12], constant strain [13], dynamic loading [14], and impact loading [15] in order to evaluate gene expression changes. Most of the previous work has utilized cartilage explants. With our model, a patella is removed from the knee and the articular cartilage is maintained intact on the underlying bone. This avoids any potential changes produced by cutting the tissue free from the surface. We have used porcine articular cartilage in our model to study the progression of OA. Animal tissue is frequently used for the study of OA progression [3, 5, 7, 10, 16–18]. More specifically, porcine tissue is readily available and has often been used for both gene expression and impact studies [8, 19–22], making it an appropriate tissue for our use as a model of impact injuries and early stage OA progression.

In our previous work developing this impact injury model of OA, we maintained intact patellae in culture for up to two weeks [8]. We evaluated loading normal to the surface at only the day 14 time point. In this study we aim to evaluate the progression of early OA symptoms by measuring gene expression changes on the day of impaction and at 3, 7, and 14 days following the impaction event. In addition to the axial impaction model we utilized previously, we also evaluated a model with increased shear forces. Eighteen genes were selected including those associated with cartilage matrix, degradative enzymes and inhibitors, inflammatory response and signaling, and cell proliferation and apoptosis and evaluated in the “traditional” impaction model and the shear model, which we believe is more indicative of a clinical injury.

## 2. Materials and Methods

### 2.1. Tissue Acquisition and Preparation

Porcine patellae were sterilely removed from knee joints obtained fresh from a local slaughterhouse. A total of 72 paired patellae were included (36 right, 36 left). The patellae were cleaned of soft tissue and the cartilage was maintained intact on the surface for testing. Throughout the testing the patellae were kept immersed in PBS with antibiotics to minimize chance of infection and prevent drying of the articular surface.

### 2.2. Impaction, Culture, and Specimen Collection

The patellae were randomized to one of three treatments: axial impaction, shear impaction, or nonimpacted control. A custom mold was used to position each patella in a test fixture on the base of a servo-hydraulic load frame for testing of the impact specimens. This allowed the patellar facet to be aligned perpendicular to the loading direction. The impactor tip was hemicylindrical and was 10 mm long by 10 mm in diameter. It was also pinned along one axis to allow rotation to accommodate any unevenness in the patellar surface. The impactor tip was attached to a piezoelectric load cell that allowed for measurement of forces in three dimensions. The axial impaction delivered a targeted load of 2000 N at 25 mm/sec [8]. The shear impaction type delivered both normal loads and an elevated shear force. This was achieved by slowly loading the articular surface to 500 N at 0.05 mm/sec. When the targeted normal loading was reached, the patella was displaced tangentially 10 mm at 200 mm/sec (via a cable and pulley system attached to a second hydraulic load frame) to induce larger shear forces.

Upon completion of the impaction, the patellae were placed into culture (Delbecco's MEM/Ham's F12 with 10% fetal calf serum, ascorbic acid (25 *μ*g/mL) with penn. 100 units/mL, strep. 100 *μ*g/mL, and amphotericin B 25 *μ*g/mL) at 37°C with 5% CO_2_ in dishes that allowed complete immersion of the patella. Culture media were changed daily to minimize chance of infection.

After culture for 0, 3, 7, or 14 days, a full-thickness cartilage specimen was harvested from the patella directly below the location of the impact and immediately flash-frozen in liquid N_2_ and stored at −80°C. The day 0 sample was collected at approximately 2 hours after impaction.

### 2.3. Gene Expression Analysis

The cartilage specimens were ground to a fine powder in a liquid nitrogen cooled mortar and pestle, and total RNA was extracted via Tri Reagent (Molecular Research Center Inc., Cincinnati, OH) following the method previously described [23]. The purity of the RNA was measured and quantitated on a Nanodrop-1000 spectrophotometer (Thermo Scientific, Wilmington, DE). A High Capacity cDNA Reverse Transcription Kit (Applied Biosystems Inc., Foster City, CA) was used for reverse transcription of 250 ng of total RNA. A panel of 18 genes related to the progression of early stage OA was evaluated in the extracted RNA. The selected genes were as follows: (1)* cartilage matrix: Col1a1, Col2a1, Acan, Sox9, Opn, *and* Comp; *(2)* degradative enzymes and inhibitors: Mmp1, Mmp3, Mmp13, Timp1, Timp2, *and* Adamts5; *(3)* inflammatory response and signaling: Ihh, Tgfb, Inos, *and* Chi3l1; *and (4)* apoptosis: Casp8, Fas* (full gene names in Table 1).

Primer pairs for quantitative real-time PCR (qPCR) were designed with Beacon Designer software (Premier Biosoft International, Palo Alto, CA) for compatibility with SYBR Green I Master Mix. When possible, primers were designed from porcine gene sequences. If not available, they were designed from conserved regions of human, bovine, or canine sequences. The primers were designed to cross an intron-exon boundary (Table 1). qPCR was performed in a 20 *μ*L reaction, consisting of 1 *μ*L of diluted cDNA, 400 nM of forward and reverse primers, 10 nM of fluorescein (as a reference dye), and 0.5 *μ*L of 1x Power SYBR Green I Master Mix. A three-step amplification protocol was performed in an iCycler iQ (Bio-Rad, Hercules, CA) with the following steps: denaturation with one cycle at 95°C for 7 minutes followed by 40 cycles of 30 sec at 95°C for denaturation, 30 sec at 50°–62°C for annealing, extension for 30 sec at 72°C, and a product melting cycle of 5 min at 72°C, 1 min at 95°C, and 1 min at 55°C. Samples were amplified in triplicate, and reaction efficiency for each primer set was assessed using standard curves via a dilution series using iCycler iQ Real-Time PCR Detection System Software. The gene target specificity of the reactions was evaluated with a melt curve generated at the end of the PCR amplification cycle. Additionally, one cDNA product from each primer pair was sequenced to verify that the PCR product corresponded to the intended gene [23]. The expressions for the genes of interest were normalized to the geometric mean of 4 reference genes identified as being the most stable in our tissue subjected to our treatment regimen [23]:* Actb, Gapdh, Sdha, *and* Ppia *[23].

### 2.4. Analysis of Results

qPCR data for the genes were evaluated by comparing the relative gene expression levels (Ct values) across treatments and across time. A linear mixed model was used for analysis following the methods proposed by Steibel et al. [24]. Differences for comparisons of interest were evaluated for statistical significance using PROC MIXED in SAS 9.2 statistical software (SAS Institute Inc., Cary, NC). The raw *P* values were adjusted for multiple comparisons using the false discovery rate (FDR) method [25]. Due to the relatively small number of samples in each combination of treatment and time in this experiment, the threshold for a significant FDR adjusted *P* value (*q*-value) was set at* q* < 0.2. This threshold allows for an appropriate sensitivity for the analyses being conducted and insures that the interpretation of the data is not overly restrictive in eliminating potentially valuable findings that may not achieve a higher level of significance. Due to the fundamental difference in how FDR controls for a type I error rate within results already deemed significant, a higher threshold may be acceptable, up to even 0.5 [26].

For each comparison of groups (example: comparing day 0 shear specimens to day 0 control specimens) the fold changes were calculated using the method of Steibel et al. [24]. Fold changes for the targeted genes of interest were normalized to the geometric mean of the previously identified four housekeeping genes. Differential gene expression between groups was first evaluated by comparing each treatment (axial versus control, shear versus control, and shear versus axial) at each time point. Differential gene expression was next evaluated within each treatment (control, axial, and shear) by comparing each time point (days 0, 3, 7, and 14) to day 0 control specimens. Day 0 control specimens were used as the reference for temporal changes as they most closely represent a cartilage surface in its natural state that has not been impacted.

## 3. Results

There were 72 patellae included in the analysis (36 right and 36 left). The patellae were randomized for treatment and collection time point. Therefore there were 6 patellae at each combination of treatment and time point (3 treatments × 4 time points × 6 patellae = 72 total patellae). RNA was extracted from one facet of each of the 72 patellae for a total of 72 specimens.

Fold changes (FC; Table 2) were evaluated within each treatment for all 18 genes over time. All genes showed significantly different (*q* < 0.2) expression within the control treatment at d14 compared to d0 with the exception of* Fas*. The most highly upregulated genes at d14 for control specimens were* Col1a1*,* Mmp1*, and* Mmp13*. The genes that have the greatest decrease in expression at d14 were* Col2a1*,* Sox9*,* Comp*, and* Casp8*. The axial specimens compared to d0 control all showed significantly different expression at d14 with the exception of* Opn*,* Ihh*, and* Inos*. Again,* Col1a1*,* Mmp1*, and* Mmp13* had the highest increase in expression, and* Col2a1*,* Sox9*,* Comp*, and* Mmp13* demonstrated the largest decreases in expression. Similar changes were observed for the shear specimens over time, with the exception that* Chi3l1* also demonstrated a large increase in expression (FC = 13.30).

**Figure 1:**
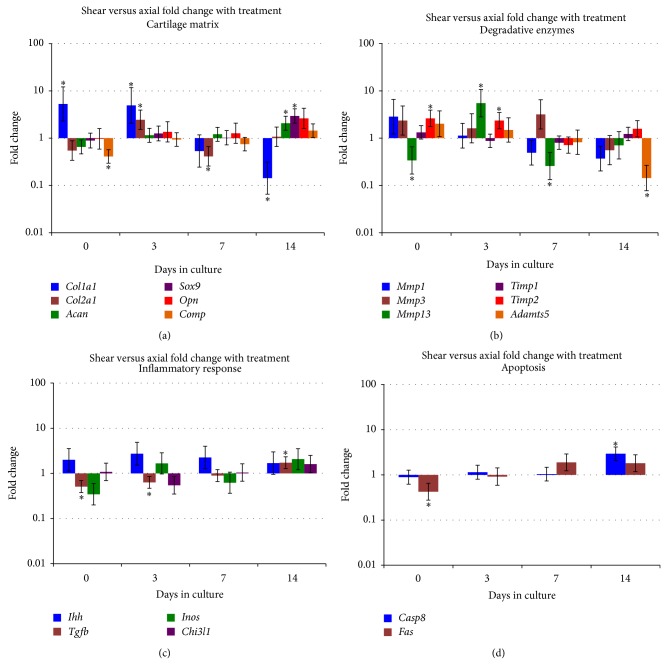
Fold changes for shear versus axial specimens within each time point. Fold change is shown on the vertical axis as a log scale, and days in culture are shown on the horizontal axis. Error bars for fold changes are depicted and significant differences for the respective comparison are indicated with an asterisk (∗). A graph is shown for each gene category: cartilage matrix (a), degradative enzymes (b), inflammatory response (c), and apoptosis (d).

Expression changes were also evaluated between treatments at each time point. The comparison of most interest was the shear impact treatment compared to the axial impact treatment (Table 2 and Figure 1).* Col1a1* had significantly higher expression at both d0 and d3 in shear versus axial specimens; however, by d14 expression was lower in shear specimens (Figure 1(a)).* Col2a1* expression was lower in shear specimens at all time points with the exception of d3 where it was 2.46-fold higher (Figure 1(a)). Both* Acan* and* Sox9* demonstrated significantly higher expression at d14, while* Comp* had significantly lower expression in shear specimens at d0 (Figure 1(a)). The* Mmp* levels were generally elevated at the earlier time point in shear compared to axial specimens; however* Mmp13* was lower at d0 (Figure 1(b)). Aggrecanase expression,* Adamts5*, was significantly lower at d14 (Figure 1(b)).* Tgfb* was the only inflammatory response gene that demonstrated significant differences in shear versus axial specimens and it showed lower expression at d0 and d3, but higher expression (FC = 1.72) at d14 (Figure 1(c)). Finally, for the apoptosis genes,* Casp8* had significantly higher expression in shear compared to axial specimens at d14 (Figure 1(d)).* Fas* also had higher expression at d14, though it was not significant (Figure 1(d)).

The fold change comparisons between the shear impact treatment compared to the nonimpacted control treatment followed similar trends to the shear treatment compared to the axial treatment (Table 3).

## 4. Discussion

The aim of this study was to identify differential gene expression changes in a porcine model of early stage cartilage degeneration in an impact injury OA model. An analysis of temporal changes in specimens over the 14 days following impact showed that* Col1a1*,* Mmp1*, and* Mmp13 *were generally upregulated over time in all treatments. The genes that have the lowest expression over time for all treatments were* Col2a1*,* Sox9*,* Comp*, and* Casp8*. The general trend of the temporal changes was similar for all treatments, including the nonimpacted control. It is therefore possible that removing the patella from the body and from its normal loading and subsequently placing it in culture affected gene expression similarly in all patellae. Therefore, to identify the effects of an individual treatment it was necessary to compare the treatments to each other within the time points.

### 4.1. Cartilage Matrix

*Col1a1* expression was elevated in shear compared to axial impacted specimens on both d0 and d3. The rise in expression of* Col1a1* may indicate that the chondrocytes are reverting to a more fibroblastic phenotype indicative of their attempt to initiate repairs, albeit with the incorrect collagen. This correlates with other work, where more damaging impacts resulted in elevated* Col1a1* expression [15], and with theories of dedifferentiated chondrocytes in OA progression [11, 27–30]. The overexpression of* Col1a1* has been correlated with chondrocyte hypertrophy in OA [31] and even focal chondrocyte cluster formation [32]. Sanchez-Adams et al. propose that impact or overload conditions may result in persisting dysfunctional chondrocyte responses to further loading, even after the injurious load is removed, and these changes may be a prelude to early OA [33]. This has been further suggested to result in a positive feedback loop where the proliferation of chondrocytes amplifies growth factors, bone cysts, and resulting damage to neighboring chondrocytes and the extracellular matrix [34].* Col2a1*, the most abundant collagen in articular cartilage, was also more highly expressed in the shear specimens on d3. Both* Acan* and* Sox9*, a transcription factor for* Acan* and* Col2a1*, were more highly expressed in shear compared to axial impact specimens on d14. The early downward trend of* Sox9* agrees with results found with a mechanical strain model [13], and the increased expression of both* Sox9* and* Acan* at d14 is indicative of repair efforts underway. The lower expression of* Comp* in shear compared to axial specimens may be indicative of matrix degradation [10, 35], possibly because the shear specimens experienced more damage during the loading event.

### 4.2. Degradative Enzymes

*Mmp* expression was generally elevated in shear compared to axial specimens at the earlier time points and then was downregulated at d14 for all* Mmp*s. In particular,* Mmp13* was significantly higher in shear specimens at d3 and then significantly lower in shear versus axial specimens by d7. The increase in degradative enzyme transcript levels is likely a response on the part of the chondrocytes to the damaging nature of the mechanical trauma. Early increase in* Mmp* levels is consistent with both high load models [15, 36] and early stage OA progression [9, 34].* Timp2 *levels were elevated in shear compared to axial specimens and were significantly higher at d0 and d3; however* Timp1* showed minimal differences between the treatments. Lee et al., however, found* Timp1* levels increased in the 24 hours following an injurious compression, with minimal changes in* Timp2*, though the general trends were similar [37].* Adamts5*, an aggrecanase, was elevated early and then showed significantly lower expression in shear versus axial specimens by d14. The findings for the degradative enzymes suggest that there is early, relatively higher matrix breakdown in the shear specimens following injury that tapers by the later time points.

### 4.3. Inflammatory Response and Signaling

*Ihh*, a signaling molecule associated with chondrocyte proliferation, was generally elevated in shear specimens in line with other studies of early stage OA [38, 39].* Tgfb* showed significantly lower expression at d0 and d3 and then higher expression at d14 in shear versus axial specimens.* Tgfb* may be a critical part of the inflammatory process for initiating repairs of the cartilage matrix and aiding cell proliferation, and lack of its expression may coincide with OA development [40].

### 4.4. Apoptosis

Both* Fas* and* Casp8* showed similar trends at each time point. Each gene had lower expression in shear versus axial specimens at d0; however* Fas* expression was significantly lower. At d14, expression of both genes was increased, but* Casp8* was significantly higher for shear versus axial specimens. In a study of aged rabbits with normal cartilage, Allen et al. found increases in* Casp8* and* Fas* expression believed to be a prelude to the development of OA [41]. Furthermore,* Casp8* was found to be upregulated in an OA transection model [42], while* Fas* expression has been found to have increased expression in the immediate vicinity of OA lesions [43]. The elevated level of apoptosis genes at d14 could indicate higher levels of apoptosis and may mean later loss of chondrocytes in the tissue.

The panel of 18 genes evaluated in this study was chosen based on their anticipated relationship to early stage OA progression. In previous work where we developed our impact injury model, we created a SAGE library to identify differentially expressed genes [8] and have used the findings to explore OA related gene expression changes in an axial impact injury model alone [44]. We correlated genes identified in our previous work with published literature to identify the most relevant genes to explore for early stage OA progression using qPCR. This work demonstrates that multiple genes in our panel have altered expression in our model of early stage OA. New technologies, such as RNA-Seq, may provide enhanced capability for detecting other genes related to OA progression [29, 45–48]. For example, Peffers et al. have completed recent work to identify nearly 400 genes that are differentially expressed between young and old equine cartilage with naturally occurring OA [29, 47]. This evolving technology promises to provide additional targets for further detailed exploration with qPCR.

## 5. Conclusions

The results presented here show a successful implementation of a porcine impact injury model for evaluating early stage OA progression. We generated a more complex loading model that incorporated elevated shear forces that may have more physiological relevance. In comparing this model to a standard normal loading model we found elevated levels of degradative enzymes and matrix constituents, similar to those found in naturally occurring cartilage degeneration. However one of those was* Col1a1*, an abnormal collagen for articular cartilage. It appears that the chondrocytes in the shear specimens are attempting repairs but are unable to mount a successful effort.

## Figures and Tables

**Table 1:** OA related genes. Full gene names, abbreviations, forward and reverse primer sequences, annealing temperatures, amplicon lengths, and NCBI numbers.

OA Related Genes					
Gene name	Gene abbreviation	Sequence (5′→ 3′)	Annealing temp	Amplicon length	NCBI Number
**Cartilage matrix**					
Collagen, Type I, Alpha 1	*Col1a1 *	F: CAACCGCTTCACCTACAGC	60	101	AK236626
		R: TTTTGTATTCGATCACTGTCTTGCC			
Collagen, Type II, Alpha 1	*Col2a1 *	F: GAGAGGTCTTCCTGGCAAAG	60	118	AF201724.1
		R: AAGTCCCTGGAAGCCAGAT			
Aggrecan	*Acan *	F: TGCAGGTGACCATGGCC	60	79	AF201722b
		R: CGGTAATGGAACACAACCCCT			
SRY (sex determining gene region Y) box-9	*Sox9 *	F: CAGGGCTCTGTGCTCTACTCC	60	230	NM_213843.1
		R: GGGTTACGGTCTTTCTTCGGT			
Osteopontin	*Opn *	F: CCGCAGCCAGGAGCAGTC	55	214	NM_214023.1
		R: GTTGATCTCAGAAGACGCACTCTC			
Cartilage oligometric matrix protein	*Comp *	F: GGCTGGAAGGACAAGACATC	55	82	XM_003123529.1
		R: CCTCATAGAACCGCACTCTG			

**Degradative enzymes & inhibitors**					
Matrix metalloprotease-1	*Mmp1 *	F: TGATGGACCTGGAGGAAACC	59	131	NM_001166229
		R: GAGCAGCCACACGATACAAG			
Matrix metalloprotease-3	*Mmp3 *	F: GATGTTGGTTACTTCAGCAC	50	197	NM_001166308.1
		R: ATCATTATGTCAGCCTCTCC			
Matrix metalloprotease-13	*Mmp13 *	F: CCAAAGGCTACAACTTGTTTCTTG	60	77	AF069643
		R: TGGGTCCTTGGAGTGGTCAA			
TIMP metallopeptidase inhibitor-1	*Timp1 *	F: CCTCGTACCAGCGTTATG			
		R: CGTTCCACAGTTGTCCAG	59	177	NM_213857.1
TIMP metallopeptidase inhibitor-2	*Timp2 *	F: ATATACGAGAACACCAGACC			
		R: GGAATGATTACAACGGATGC	59	152	AK237154.1
ADAM metallopeptidase with thrombospondin Type 1 motif 5	*Adamts5 *	F: CGCTGCCACCACACTCAA R: CGTAGTGCTCCTCATGGTCATCT	60	80	NM_007038.3

**Inflammatory response**					
Indian hedgehog	*Ihh *	F: CAGCGGGCGCTATGAAGGCA	60	140	XM_001925486.1
		R: GGTCCTTGCAGCGCTGGGTC			
Transforming growth factor *β*	*Tgfb *	F: GGAGTGGCTGTCCTTTGATGT	60	117	NM_214015.1
		R: AGTGTGTTATCTTTGCTGTCA			
Nitric oxide synthase 2, inducible	*Inos *	F: TGAATTTGTCAACCTGTATTAC	53	82	NM_001143690.1
		R: CTTTGTTACCGCTTCCAC			
Chitinase-3-like protein 1	*Chi3l1 *	F: TGACGCTCTATGACACAC	62	194	NM_001001540
		R: GGCTAGGTCCAGTCCATC			

**Cell proliferation & apoptosis**					
Caspase-8	*Casp8 *	F: TGGGCAAACAGATGCCACAACCT	60	153	NM_001031779.2
		R: CCCCTTCAATCTAGCCCACCCCC			
*Fas* (TNF receptor superfamily, member 6)	*Fas *	F: TAGAGTTTGTGATGGAGAA	53	107	NM_213839.1
		R: ATTGAGAAGTGTGACAGA			

**Table 2:** Differential gene expression for shear compared to axial specimens at each time point. Fold changes are shown on the left, and the corresponding *q*-values are shown on the right. Significant *q*-values (*q* < 0.2) and the associated fold changes are in bold.

Comparing treatments within time point								
Genes grouped by functional type	Fold changes				*q*-values (FDR)			
	Shear versus axial				Shear versus axial			
	Day 0	Day 3	Day 7	Day 14	Day 0	Day 3	Day 7	Day 14
Cartilage matrix								
* Col1a1 *	**5.29**	**4.93**	0.54	**0.14**	**0.09**	**0.09**	0.43	**0.05**
* Col2a1 *	0.55	**2.46**	**0.41**	1.07	0.27	**0.12**	**0.12**	0.88
* Acan *	0.66	1.15	1.21	**2.07**	0.43	0.68	0.68	**0.13**
* Sox9 *	0.89	1.26	1.02	**2.95**	0.95	0.95	0.95	**0.01**
* Opn *	0.97	1.36	1.27	2.63	0.95	0.83	0.83	0.20
* Comp *	**0.41**	0.94	0.75	1.44	**0.03**	0.84	0.51	0.51
Degradative enzymes & inhibitors								
* Mmp1 *	2.84	1.13	0.49	0.37	0.32	0.84	0.32	0.32
* Mmp3 *	2.36	1.62	3.19	0.56	0.45	0.50	0.41	0.50
* Mmp13 *	**0.34**	**5.51**	**0.26**	0.71	**0.14**	**0.05**	**0.08**	0.60
* Timp1 *	1.33	0.88	0.80	1.23	0.69	0.69	0.69	0.69
* Timp2 *	**2.63**	**2.36**	0.71	1.58	**0.06**	**0.06**	0.39	0.33
* Adamts5 *	2.02	1.49	0.82	**0.14**	0.52	0.66	0.74	**0.01**
Inflammatory response & signaling								
* Ihh *	2.00	2.73	2.25	1.68	0.30	0.30	0.30	0.36
* Tgfb *	**0.51**	**0.63**	0.89	**1.72**	**0.11**	**0.18**	0.72	**0.15**
* Inos *	0.35	1.67	0.62	2.06	0.21	0.38	0.38	0.36
* Chi3l1 *	1.08	0.54	1.05	1.61	0.92	0.57	0.92	0.57
Cell proliferation & apoptosis								
* Casp8 *	0.89	1.15	1.04	**2.95**	0.91	0.91	0.91	**0.01**
* Fas *	**0.43**	0.92	1.90	1.81	**0.19**	0.85	0.22	0.22

**Table 3:** Differential gene expression for shear compared to control specimens at each time point. Fold changes are shown on the left, and the corresponding *q*-values are shown on the right. Significant *q*-values (*q* < 0.2) and the associated fold changes are in bold.

Comparing treatments within time point								
Genes grouped by functional type	Fold changes				*q*-values (FDR)			
	Shear versus control				Shear versus control			
	Day 0	Day 3	Day 7	Day 14	Day 0	Day 3	Day 7	Day 14
Cartilage matrix								
* Col1a1 *	1.37	**5.36**	**0.17**	0.35	0.70	**0.09**	**0.09**	0.24
* Col2a1 *	1.41	0.97	1.13	1.96	0.94	0.94	0.94	0.62
* Acan *	0.82	1.29	1.14	1.53	0.70	0.70	0.70	0.70
* Sox9 *	1.00	0.71	0.62	1.33	0.99	0.56	0.56	0.56
* Opn *	1.02	2.26	0.78	0.58	0.97	0.40	0.83	0.53
* Comp *	0.60	0.87	0.93	0.81	0.47	0.83	0.83	0.83
Degradative enzymes & inhibitors								
* Mmp1 *	1.55	0.67	0.39	**0.17**	0.60	0.60	0.25	**0.01**
* Mmp3 *	**3.69**	0.61	**3.58**	**0.26**	**0.10**	0.49	**0.10**	**0.10**
* Mmp13 *	1.05	**3.56**	0.54	**0.19**	0.94	**0.12**	0.47	**0.05**
* Timp1 *	1.24	0.85	0.96	0.84	0.84	0.84	0.90	0.84
* Timp2 *	0.96	1.34	0.49	1.03	0.93	0.93	0.28	0.93
* Adamts5 *	0.52	0.87	**0.37**	**0.22**	0.39	0.81	**0.18**	**0.04**
Inflammatory response & signaling								
* Ihh *	2.33	0.83	2.05	1.27	0.42	0.74	0.42	0.74
* Tgfb *	0.72	1.22	0.86	0.68	0.57	0.63	0.63	0.57
* Inos *	0.77	0.92	0.94	**0.31**	0.91	0.91	0.91	**0.13**
* Chi3l1 *	**2.00**	**0.46**	1.20	**0.51**	**0.17**	**0.17**	0.69	**0.17**
Cell proliferation & apoptosis								
* Casp8 *	0.98	0.70	0.63	1.31	0.95	0.58	0.58	0.58
* Fas *	**0.35**	1.20	0.84	1.45	**0.06**	0.69	0.69	0.69
